# Postnatal growth in preterm infants during the first year of life: A population-based cohort study in China

**DOI:** 10.1371/journal.pone.0213762

**Published:** 2019-04-11

**Authors:** Leni Kang, Huiqing Wang, Chunhua He, Ke Wang, Lei Miao, Qi Li, Yanping Wang, Jun Zhu, Xiaohong Li, Xingzhe Liu, Jiawei Chen, Qianrun Chen, Dezhi Mu

**Affiliations:** 1 National Office for Maternal and Child Health Surveillance of China, Key Laboratory of Birth Defects and Related Diseases of Women and Children of Ministry of Education, West China Second University Hospital, Sichuan University, Chengdu, Sichuan, China; 2 Department of Pediatrics, Key Laboratory of Birth Defects and Related Diseases of Women and Children of Ministry of Education, West China Second University Hospital, Sichuan University, Chengdu, Sichuan, China; 3 West China School of Medicine, Sichuan University, Chengdu, Sichuan, China; Monash University, AUSTRALIA

## Abstract

In preterm infants (i.e. the gestational age less than 37 weeks), postnatal growth remains a concern. This study used multicenter longitudinal data from China’s Under 5 Child Nutrition and Health Surveillance System to investigate the postnatal growth in the weight and length of preterm infants. Gender-stratified differences in weight and length were assessed between preterm and term infants. 1221 preterm infants and 1221 matched term infants were included. The rates of growth in weight and length in preterm infants was greater than those in term infants, especially from the first to sixth month. The rates were higher in males compared to females in the first 3 months. The differences of weight and length between preterm and term infants decreased with increasing age, however, these measurements did not reach the level of their term peers until 12 months before adjusting for gestational age. The median values of weight and length were even larger in preterm infants in the first month after adjusting for gestational age.

## Introduction

Preterm birth complications are the leading causes of death among children aged under 5 years globally, with an estimated 965000 deaths reported in 2013 [1]. Additionally, these complications are the second largest contributors to the under 5 mortality in China [2]. In addition, preterm birth has been reported to be associated with impaired neurodevelopment [3], adverse cognitive and behavioral outcomes [4], low exercise capacity [5], and increased risk of chronic diseases, such as hypertension [6], and type 2 diabetes mellitus [7]. It has been estimated that the rate of preterm birth was 7.10% in China, amounting to approximately 1200000 preterm births in 2010 and ranking the second in the world [8].

In preterm infants, postnatal growth is always a concern. Weight and length are sensitive and convenient indices for evaluating growth in children. Studies have shown that preterm infants are lighter and shorter than same-aged term infants during childhood [9, 10], while other studies have identified a period of catch-up growth during the first few years among preterm and/or low birth weight infants [11, 12]. Recently, the INTERGROWTH-21^st^ Project established standards for postnatal growth in preterm infants that could be used to assess preterm infants until 64 weeks’ postmenstrual age [13].

However, little is known about the early postnatal growth patterns of preterm infants in China. Therefore, we analyzed data from a population-based surveillance system (i.e., longitudinal study) to compare growth patterns of weight and length in preterm infants within the first year with their term peers.

## Materials and methods

### Data source

Data from China’s Under 5 Child Nutrition and Health Surveillance System (U5CNHSS) were used in this study. U5CNHSS is a population-based dynamic surveillance system launched by the Chinese government in 2011. The system collects data from a representative sample of 80 districts/counties (including 47 districts in the urban area and 33 counties in the rural area) across 31 provinces/autonomous regions/municipalities (excluding Hong Kong, Macao, and Taiwan). Then, the number of villages/residential committees in each township/street of the sampled district/county was ranked, and four townships/streets are randomly selected using a systematic sampling method. For each selected township/street, several villages/residential committees were selected until a minimal sample size of 500 under 5 children was reached. This study was approved by the Ethics Committee of West China Second University Hospital, Sichuan University, China.

### Subjects

**Fig 1:**
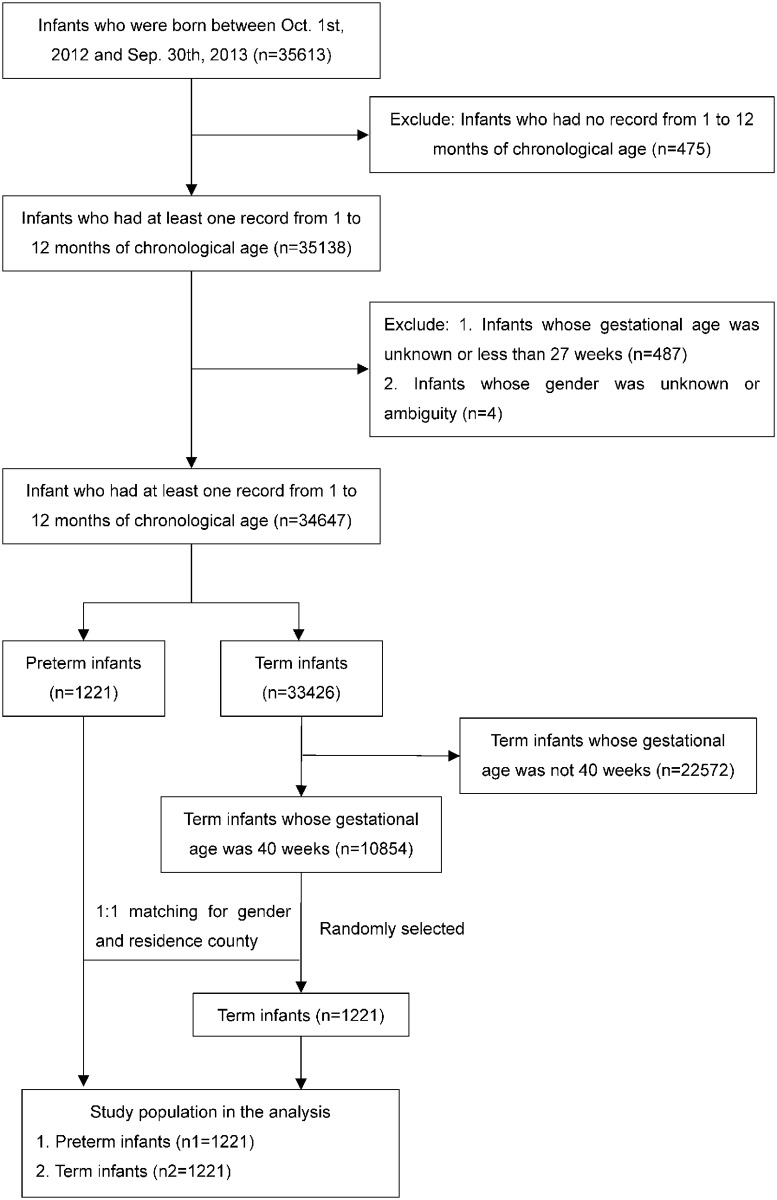
The flow chart of the study population selection.

The inclusion criteria for this analysis is as follows: 1) infants who were born between Oct. 1^st^, 2012 and Sep. 30^th^, 2013; 2) infants who had at least one record of weight and length measurement during the period from 1 to 12 months of chronological age; and the exclusion criteria is as follows: 1) infants whose gestational age was unknown or less than 27 weeks; 2) infants whose gender was unknown or ambiguity. Then, the study population were further separated into preterm and term infants. All preterm infants who met the inclusion criteria were included. Term infants with gestational age of 40 weeks and 1:1 matched by gender and residence place at the county level (i.e. the same county with the preterm infants) were randomly selected. Since there was no information on the genetic and chromosomal disorders of these subjects, we could not exclude those infants. The flow chart of the study population selection was shown in Fig 1.

### Data collection

Unified protocol and forms were used for each surveillance site. Village or community doctors were obligated to register all newborns and under 5 children within their responsible areas and recruit them for health examinations in the township or community health care center. Infants were followed up at 1-, 3-, 6-, 8-, 12-months of chronological age. The health examination included weight and length measurements, a basic physical examination, growth and developmental evaluation, and a questionnaire consisting of questions regarding the demographic information for the household, mother and child; and medical conditions for the mother and child. Gestational age was based on the last menstrual period of the mother and/or first-trimester ultrasonography. The weight and length measurements were obtained by the trained health workers according to the nationwide technical specification of essential public health service [14]. Weight and length were measured to 0.01 kg and 0.10 cm, respectively. The data were then entered into the network report system within one month after each visit, and a level-by-level audit and routine quality control were performed.

### Quality control

The village or community doctors checked the list of newborns and under 5 children monthly and ensured that each child finished his or her health visit. Health workers in the township or community health care centers double checked the list of children who finished health visits with the village or community doctors and checked the completeness of the forms. Health workers in the county/district-, prefecture-, provincial-, and national-level sampled 2 to 3 surveillance sites semiannually or annually for quality control concerning the workflow, measuring method, instrument adjustment record and data quality at each site.

### Statistical analysis

**Fig 2:**
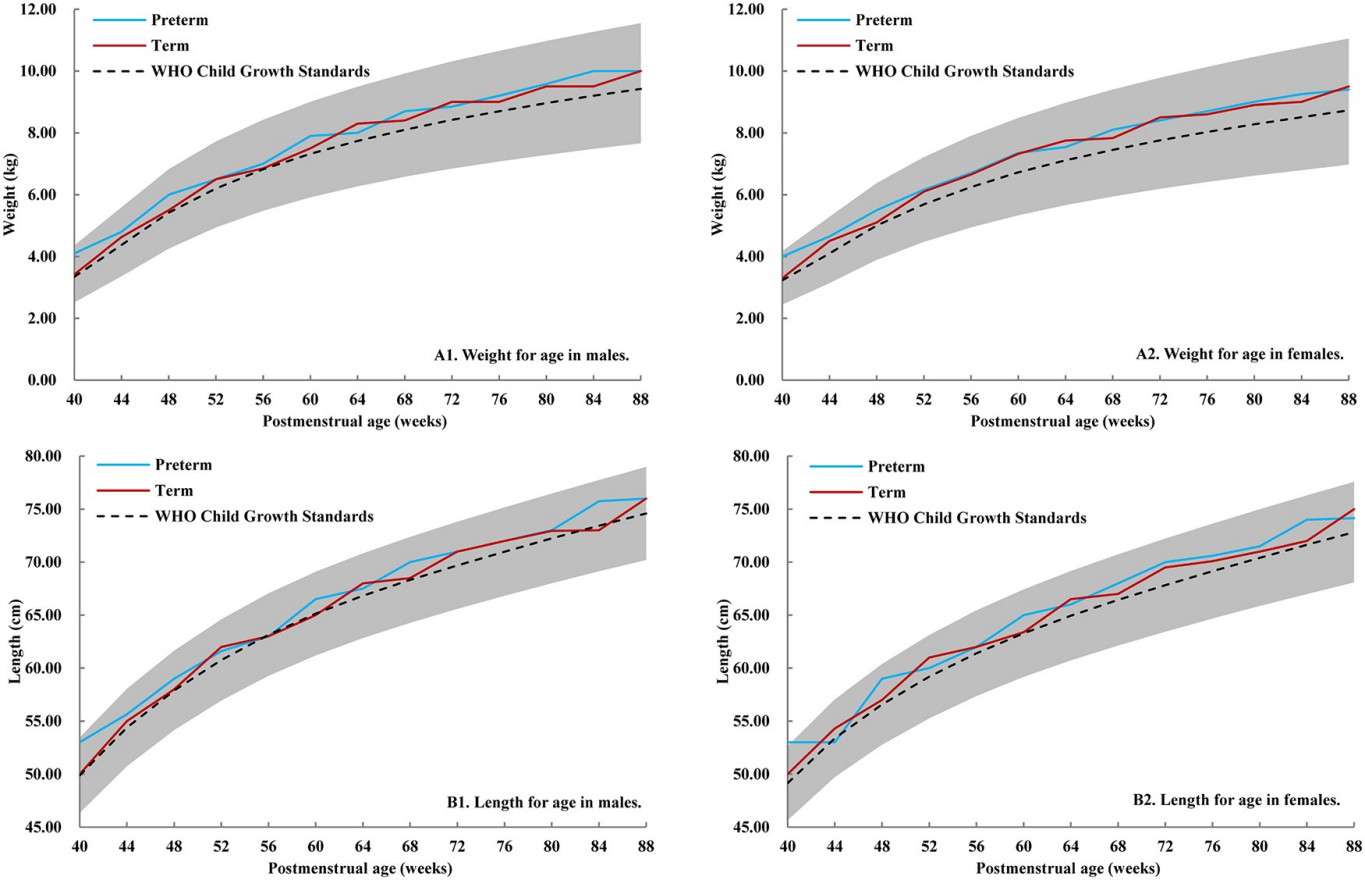
The trajectories of weight and length in preterm and term infants over time, by postmenstrual age. **(A1) Weight for age in males. (A2) Weight for age in females. (B1) Length for age in males. (B2) Length for age in females**. All lines are represented to median values. The blue line is the trajectory of preterm infants, the red line is the trajectory of term infants, the black dotted line is the trajectory of WHO Child Growth Standards. The gray area is 3^rd^ to 97^th^ centiles for weight and length of WHO Child Growth Standards.

**Fig 3:**
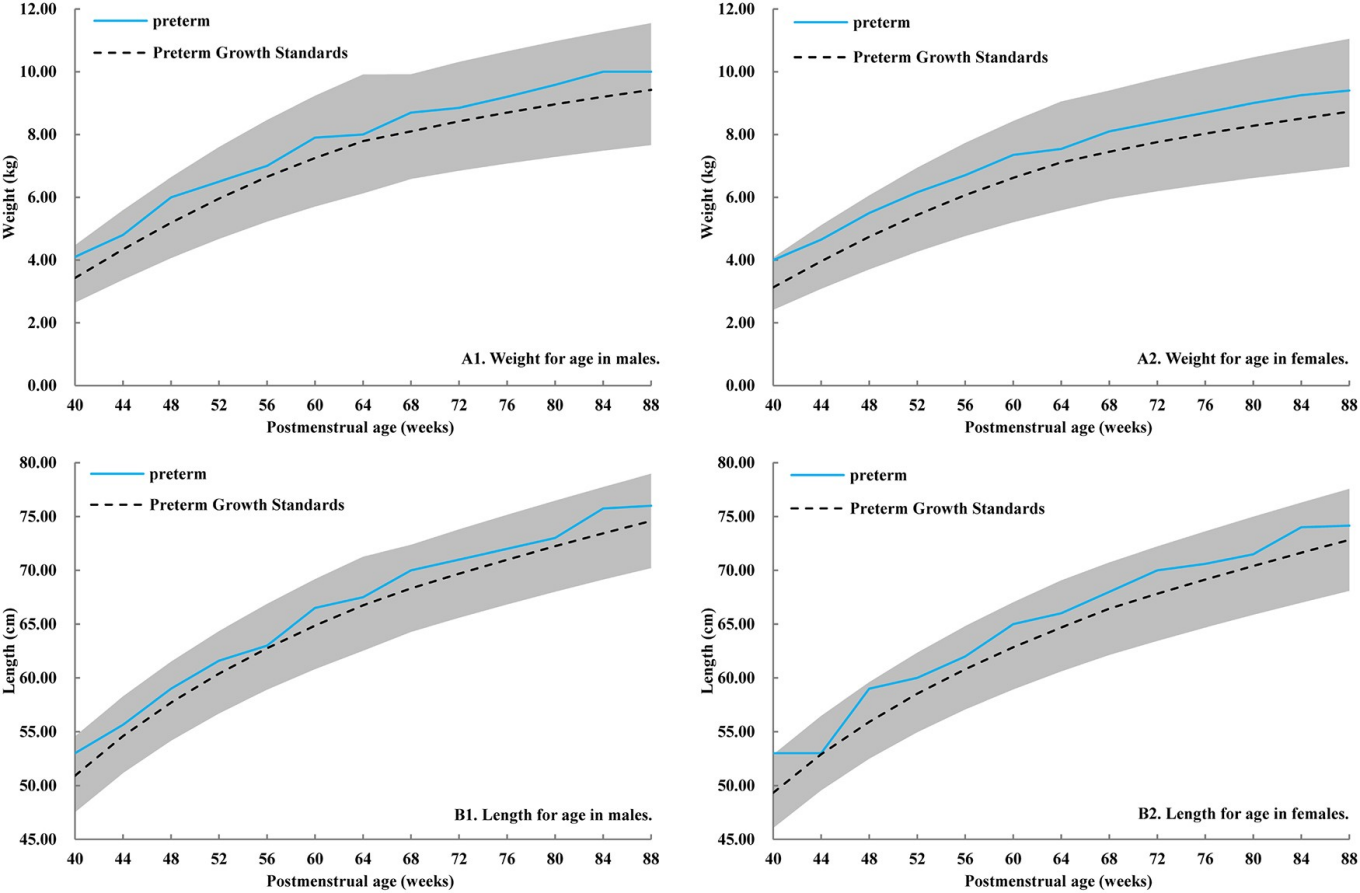
The trajectories of weight and length in preterm infants over time, by postmenstrual age. **(A1) Weight for age in males. (A2) Weight for age in females. (B1) Length for age in males. (B2) Length for age in females**. All lines are represented to median values. The blue line is the trajectory of preterm infants, the black dotted line is the trajectory of postnatal growth standards for preterm infants. The standards were combined with two parts, the INTERGROWTH-21^st^ Preterm Postnatal Growth Standards were used from 40 to 64 postmenstrual weeks, and the WHO Child Growth Standards were used from 65 to 88 postmenstrual weeks. The gray area is 3^rd^ to 97^th^ centiles for postnatal weight and length of infants in preterm growth standards.

Preterm infants were defined as those that were born at a gestational age less than 37 weeks. In this study, both chronological age (i.e., postnatal age) and corrected age (i.e., correct to 40 gestational weeks) were used for preterm infants. Postmenstrual age was used for comparison in Figs 2 and 3. Fig 3 used INTERGROWTH-21^st^ Preterm Postnatal Growth Standards from 40 to 64 postmenstrual weeks, and used WHO Child Growth Standards from 65 to 88 postmenstrual weeks. The measurements were included in the analysis only if the physical exam time were within 10% days of the specific time points (either before or after adjusting). The size for gestational age was accessed according to China’s neonatal birth weight curve [15]. The weights and lengths of males and females were expressed as the mean standard deviation (SD) for preterm and term infants, respectively. The differences for qualitative data and quantitative data between preterm and term infants were assessed by Chi-square test and T-test, respectively.

All statistical analyses were performed by SAS 9.4 software (SAS Institute Inc., Cary, NC, USA). The statistical significance level for α was set at 0.05.

## Results

A total of 2442 infants were included in the analysis, of which 1221 were born as premature. The average gestational age of preterm infants were 35.04±1.43 weeks (range: 27–36 weeks), and most of the preterm infants (76.09%) were born at greater than 34 gestational weeks. A larger proportion of preterm infants were evaluated as small for gestational age (SGA) compared to term infants (10.07% vs. 7.94%). The average maternal age at delivery was greater in the mothers of preterm infants than the mothers of term infants (29.12±5.06 vs. 27.94±4.40, *p*<0.001). A higher proportion of preterm infants were exclusive breastfed compared to term infants (5.03% vs. 2.72%). No differences were observed in maternal education level and migrant status between the two groups. (Table 1).

**Table 1:** Baseline characteristics of infants according to gestational age.

	Preterm		Term		P value
	N	Col%	N	Col%	
**Number of participants**	1221	-	1221	-	-
**Gestational age (weeks)**					
27–31	40	3.28	-	-	-
32–34	252	20.64	-	-	
35–36	929	76.09	-	-	
**Infants’ auxologic parameters**					
Small for Gestational Age (SGA)	123	10.07	97	7.94	0.002
Appropriate for Gestational Age (AGA)	953	78.05	1020	83.54	
Large for Gestational Age (LGA)	145	11.88	104	8.52	
Maternal education level*					
Primary school and below	46	3.79	42	3.45	0.783
Junior middle school	405	33.36	403	33.06	
Senior high school / technical secondary school	266	21.91	287	23.54	
Junior college or above	497	40.94	487	39.95	
**Maternal age at delivery (Mean±SD)**	29.12±5.06		27.94±4.40		<0.001
**Migrants**					
No	1122	91.89	1107	90.66	0.282
Yes	99	8.11	114	9.34	
Feeding mode*					
Exclusive breastfeeding	50	5.03	28	2.72	0.007
Formula milk with breastfeeding	463	46.53	469	45.53	
Formula milk without breastfeeding	39	3.92	27	2.62	
Others	443	44.52	506	49.13	

Note:

*, there were missing data.

Table 2 showed the differences in weight and length between preterm and term infants, stratified by gender. Generally, the median values for weight and length in term infants were consistently larger than preterm infants during the first year in both gender groups when using chronological age, but the differences decreased with increasing age. After adjusting for gestational age, the median values for weight and length were significantly greater in preterm infants at the corrected birthday in both genders. These differences also decreased with increasing age, and term infants even turn the tide from the sixth month. The weight and length for preterm infants in both genders showed no differences before and after adjusting until 12 months.

**Table 2:** Postnatal growth of weight (kg) and length (cm) in preterm and term infants.

Gender	Postnatal age (months)	Weight (Median, IQR)					Length (Median, IQR)				
		Preterm (1)	Preterm-a (2)	Term (3)	Δ1 (3)-(1)	Δ2 (3)-(2)	Preterm (1)	Preterm-a (2)	Term (3)	Δ1 (3)-(1)	Δ2 (3)-(2)
**Males**	0	2.55 (2.20, 2.90)	4.10 (3.60, 4.50)	3.42 (3.20, 3.70)	0.95* (0.45, 1.40)	-0.71* (-1.20, -0.10)	48.00 (46.00, 50.00)	53.00 (52.00, 55.00)	50.00 (50.00, 51.00)	2.00* (0.00, 5.00)	-3.00* (-4.50, -1.00)
	1	3.80 (3.40, 4.48)	5.30 (4.80, 6.00)	4.80 (4.50, 5.00)	0.88* (0.34, 1.50)	-1.10 (-1.36, 0.10)	52.70 (51.00, 54.00)	57.00 (55.00, 58.70)	55.00 (54.00, 56.50)	2.50* (1.00, 5.00)	-2.50 (-4.00, 1.00)
	3	6.04 (5.50, 6.50)	6.75 (6.00, 7.45)	6.80 (6.20, 7.25)	0.60* (-0.01, 1.40)	-0.24 (-0.80, 0.80)	60.00 (58.00, 61.50)	62.00 (60.00–64.00)	62.20 (61.00, 64.00)	2.50* (0.50, 5.00)	-1.00 (-3.50, 2.00)
	6	8.00 (7.45, 8.50)	8.20 (7.70, 9.00)	8.30 (7.75, 9.00)	0.45* (-0.40, 1.15)	0.08 (-0.81, 0.70)	67.00 (65.00, 68.50)	68.00 (66.70–70.00)	68.30 (66.60, 70.00)	1.50* (-0.50, 4.00)	0.50 (-2.00, 2.00)
	8	8.70 (8.00, 9.40)	9.00 (8.34, 9.80)	9.00 (8.50, 9.80)	0.30* (-0.50, 1.30)	0.20 (-0.70, 1.10)	70.40 (68.80, 72.00)	71.10 (70.00–73.00)	72.00 (70.00, 73.00)	1.00* (-1.00, 3.50)	0.50 (-1.50, 2.20)
	12	10.00 (9.40, 10.75)	10.00 (9.50, 11.00)	10.10 (9.50, 11.00)	0.30* (-0.80, 1.20)	0.20 (-0.90, 1.00)	76.00 (74.00, 77.40)	76.00 (74.50–78.00)	76.20 (75.00, 78.00)	1.35* (-2.00, 3.15)	1.00* (-2.00, 3.00)
**Females**	0	2.55 (2.20, 2.90)	4.00 (3.60, 4.30)	3.30 (3.05, 3.60)	0.80* (0.32, 1.20)	-0.60* (-1.14, -0.10)	48.00 (46.00, 50.00)	53.00 (51.00–54.00)	50.00 (50.00, 50.00)	2.00* (0.00, 4.00)	-2.75* (-4.00, -1.00)
	1	3.80 (3.40, 4.20)	4.45 (4.00, 5.00)	4.50 (4.20, 4.85)	0.79* (0.10, 1.30)	0.00 (-0.50, 0.65)	52.00 (50.00, 54.00)	54.50 (52.00–55.00)	55.00 (53.10, 56.00)	3.00* (0.15, 4.40)	-1.00 (-2.00, 0.00)
	3	5.85 (5.30, 6.20)	6.30 (5.90, 6.68)	6.20 (5.90, 6.70)	0.45* (-0.15 1.00)	0.00 (-0.48, 0.63)	59.00 (57.50, 60.50)	60.00 (59.00–62.50)	61.00 (60.00, 62.00)	2.00* (0.00, 4.00)	0.00 (-1.75, 3.25)
	6	7.50 (7.00, 8.00)	7.80 (7.22, 8.50)	7.80 (7.20, 8.50)	0.39* (-0.30, 1.16)	0.10 (-0.65, 0.95)	65.50 (64.00, 67.00)	66.50 (65.00–68.50)	67.00 (65.00, 68.00)	1.50* (-0.40, 3.60)	0.25 (-1.50, 2.50)
	8	8.30 (7.70, 9.00)	8.50 (7.95, 9.10)	8.50 (8.00, 9.15)	0.30* (-0.50, 1.10)	0.10 (-0.53, 1.13)	69.50 (67.50, 71.00)	70.00 (69.00–72.00)	70.00 (68.70, 72.00)	1.00* (-1.00, 3.00)	0.50 (-1.00, 2.50)
	12	9.40 (8.72, 10.00)	9.40 (8.80, 10.00)	9.60 (9.00, 10.10)	0.27* (-0.60, 1.00)	0.25* (-0.60, 1.10)	74.00 (73.00, 76.00)	74.50 (73.00–76.00)	75.00 (74.00, 77.00)	1.00* (-1.00, 3.00)	1.00* (-1.00, 3.00)

Note: IQR, interquartile range. Preterm, weight/length for age in preterm infants, using chronological age. Preterm-a, weight/length for age in preterm infants, using corrected age. Term, weight/length for age in term infants, using chronological age. Δ1, differences of weight/length for age between term and preterm infants, using chronological age for preterm infants. Δ2, differences of weight/length for age between term and preterm infants, using corrected age for preterm infants.

*, the differences are statistically significant.

Weight and length for age in males and females for both term and preterm infants were illustrated by median values using postmenstrual age to make equitable comparisons. All measurements were within the 3^rd^ to 97^th^ centiles of 2006 WHO Child Growth Standards. Most of the median values for weight and length were greater in both term and preterm infants in our study population during the first 88 postmenstrual weeks when compared to the WHO standards. Preterm infants were even heavier and longer than their term peers at 40 postmenstrual weeks. Furthermore, we could see a rapid growth between 40^th^ and 52^th^ postmenstrual weeks in all groups. (Fig 2).

We further compared the growth pattern of preterm infants with postnatal growth standards for preterm infants in the INTERGROWTH-21^st^ study. The median values of weight and length in preterm infants increased with increasing age and within the 3^rd^ to 97^th^ centiles for preterm growth standards. Generally speaking, preterm infants in our study were heavier and longer than the standards in both genders. (Fig 3).

The rates of postnatal growth in weight and length for preterm and term infants during different time periods were listed in Table 3. The postnatal growth rates of weight and length in both genders decreased with increasing age. Generally, preterm infants consistently grew faster than term infants, especially from the first to sixth month after birth. Males grew faster than females within the first 3 months.

**Table 3:** The postnatal growth rates of weight (kg/week) and length (cm/week) for preterm and term infants during the first year.

Gender	Postnatal age (months)	Weight (Median, IQR)		Length (Median, IQR)	
		Preterm	Term	Preterm	Term
**Males**	0–1	0.27^#^ (0.21, 0.35)	0.28^#^ (0.22, 0.34)	1.00*^#^ (0.70, 1.28)	1.09^#^ (0.85, 1.35)
	1–3	0.24*^#^ (0.20, 0.30)	0.22^#^ (0.18, 0.27)	0.82*^#^ (0.69, 0.99)	0.79^#^ (0.65, 0.93)
	3–6	0.14* (0.11, 0.17)	0.11 (0.09, 0.15)	0.54* (0.46, 0.62)	0.46^#^ (0.37, 0.54)
	6–8	0.10 (0.06, 0.14)	0.08 (0.05, 0.12)	0.42 (0.31, 0.55)	0.40 (0.28, 0.53)
	8–12	0.07 (0.04, 0.09)	0.06 (0.04, 0.09)	0.29* (0.24, 0.35)	0.27 (0.22, 0.32)
**Females**	0–1	0.26 (0.20, 0.34)	0.26 (0.21, 0.31)	0.90 (0.68, 1.24)	1.00 (0.72, 1.27)
	1–3	0.21* (0.17, 0.26)	0.20 (0.16, 0.24)	0.78* (0.64, 0.92)	0.73 (0.57, 0.87)
	3–6	0.13* (0.10, 0.16)	0.11 (0.09, 0.14)	0.49* (0.40, 0.59)	0.44 (0.36, 0.51)
	6–8	0.09 (0.06, 0.13)	0.09 (0.06, 0.12)	0.42* (0.34, 0.57)	0.38 (0.29, 0.49)
	8–12	0.06 (0.04, 0.08)	0.06 (0.04, 0.08)	0.29 (0.23, 0.34)	0.30 (0.23, 0.35)

Note: IQR, interquartile range.

*, Comparison between preterm and term infants with statistically significant results, before adjusting for gestational age.

^#^, Comparison between males and females with statistically significant results, before adjusting for gestational age.

## Discussion

In this study, we compared postnatal growth in weight and length during the first year between preterm and term infants. The preterm birth rate identified in this study was 3.53%, which is less than global estimates [8] but comparable with those reported in another study conducted in China (3.72%) [16]. This study was based on community/village source (a convenience sample), and preterm infants (especially those extremely preterm infants) were less likely to be included in the routine physical examinations.

We noted that after adjusting for gestational age, preterm infants were consistently heavier and taller than the WHO Child Growth Standards for both males and females, and these measurements for term infants were similar to or larger than the WHO standards. This could be interpreted by the “One Child” policy in China conducted before 2016. Parents and/or other caregivers devote more time and efforts to their only child in the family. They believe the heavier and taller the child is, the healthier the child is. Many studies in China have also reported that Chinese infants are significantly heavier and longer than the age-specific WHO growth standards recommended [17–19].

We found that the rates of growth after birth were more rapid in preterm infants, especially from the third to sixth month, and the rates were higher in males than females within the first 3 months. That is, preterm infants showed catch-up growth for some of the deficits in weight and length after birth, and males caught up even faster. This could also explain the phenomenon that preterm infants were even heavier and longer than term infants in the first month after adjusting for gestational age. This finding could be verified by Gong et al.’s study, and they reported even overweight/over-length in preterm infants at a 10th/11th month. The INTERGROWTH-21^st^ Project discovered that the patterns differed between postnatal growth and intrauterine growth [13]. When compared to the Chinese neonatal birth weight curve [15], the weight increased from 1053 g at 27 gestational weeks to 3482 g at 40 gestational weeks (i.e. 0.187 kg per week), which was lower than the changes in preterm infants from born to the third month.

However, these differences decreased with increasing age and term infants even turn the tide after the sixth month. The weight and length of both males and females in preterm infants did not achieve the median measurements of their term peers. Zhao et al. [20] found a faster growth rate in preterm infants during the first 12 months of age with the growth rate peaking at 1–3 months of age, but not yet catch up with term infants. Another study from China reported the catch-up growth occurred from 10th and 12th month for weight and length, respectively [21]. We believe that the rapid growth in preterm infants could be explained by the parents of preterm infants giving their child more care and nutritional supplements to make up the backward growth at birth. This could be also partly proved by our data that a higher proportion of preterm infants acquired breastfeeding by their mothers. Interestingly, in the first month, preterm infants did not show catch-up growth in both weight and length. This may because of the physiological characteristics of preterm infants. Their lack of intrauterine stockpiles and immature gastrointestinal functions made them poor digestion and absorption, and increased stress metabolism [22].

Studies have reported that catch-up growth could reduce the rate of later stunting and be protective against infection in early life [23]. Nevertheless, an increasing number of studies have reported an association between adverse long-term outcomes and faster growth in infancy. Reviews have shown that catch-up growth during the first few months of life can lead to obesity, cardiovascular disease, and type 2 diabetes mellitus in adulthood [24–27]. Thus, whether and how catch-up growth should be promoted, especially in low-income settings, is a question that needs to be addressed. Another review recommended that in developing countries, promotion of catch-up growth beyond the first 3 months of life may be safe and appropriate [17]. In China, more attentions have been taken to enhance the growth potential of preterm infants, however, scientific feeding is still on the way.

We acknowledged several limitations. First, this study was limited by the study population, infants died within the first month of age could not be included in our study. As is known, preterm infants and infants with low birth weight may be more likely to die during the first month of life than their term peers. Our study may underestimate the rate of preterm birth and overestimate the weight and length of infants, especially preterm infants. Second, since the time point of physical measurements were based on the chronological age, the measurements at the adjusted age for preterm is limited, which made further comparison difficult. Third, due to the study endpoint, this study could not determine whether and when the weight and length of both genders, caught up with their term peers.

## Conclusions

Preterm infants grew faster than term infants from the first to sixth month after birth. The differences in weight and length between preterm and term infants decreased with increasing age. However, preterm infants did not reach the median values of weight and length for term infants during the first year when using the chronological age.
